# Expression of Groucho/TLE proteins during pancreas development

**DOI:** 10.1186/1471-213X-8-81

**Published:** 2008-09-08

**Authors:** Brad G Hoffman, Bogard Zavaglia, Mike Beach, Cheryl D Helgason

**Affiliations:** 1 Department of Cancer Endocrinology, BC Cancer Research Center, 675 West 10th Avenue, Vancouver, B.C., V5Z 1L3, Canada; 2 Department of Surgery, Faculty of Medicine, University of British Columbia, 910 West 10th Avenue, Vancouver, B.C., V5Z 4E3, Canada

## Abstract

**Background:**

The full-length mammalian homologs of *groucho*, Tle1, 2, 3, and 4, act as transcriptional corepressors and are recruited by transcription factors containing an eh1 or WRPW/Y domain. Many transcription factors critical to pancreas development contain a Gro/TLE interaction domain and several have been shown to require Gro/TLE interactions for proper function during neuronal development. However, a detailed analysis of the expression patterns of the Gro/TLE proteins in pancreas development has not been performed. Moreover, little is known about the ability of Gro/TLE proteins to interact with transcription factors in the pancreas.

**Results:**

We describe the expression of Gro/TLE family members, and of 34 different transcription factors that contain a Gro/TLE interaction motif, in the pancreas utilizing nine SAGE libraries created from the developing and adult pancreas, as well as the *GenePaint *database. Next, we show the dynamic expression of *Tle1*, *2*, *3*, *4, 5 *and *6 *during pancreas development by qRT-PCR. To further define the cell-type specificity of the expression of these proteins we use immunofluorescence to co-localize them with Pdx1 at embryonic day 12.5 (E12.5), Ngn3 at E14.5, Pdx1, Nkx2-2, Insulin, Glucagon, Pancreatic polypeptide and Somatostatin at E18.5, as well as Insulin and Glucagon in the adult. We then show that Tle2 can interact with Nkx2-2, Hes1, Arx, and Nkx6-1 which are all critical factors in pancreas development. Finally, we demonstrate that Tle2 modulates the repressive abilities of Arx in a β-cell line.

**Conclusion:**

Although Tle1, 2, 3, and 4 show overlapping expression in pancreatic progenitors and in the adult islet, the expression of these factors is restricted to different cell types during endocrine cell maturation. Of note, Tle2 and Tle3 are co-expressed with Gro/TLE interaction domain containing transcription factors that are essential for endocrine pancreas development. We further demonstrate that Tle2 can interact with several of these factors and that Tle2 modulate Arx's repressive activity. Taken together our studies suggest that Gro/TLE proteins play a role in the repression of target genes during endocrine cell specification.

## Background

Many of the transcription factors important in pancreas development are, or are thought to act as, repressors of target genes. For example, Nkx2-2 acts as a transcriptional repressor in the developing neural tube [1,2] and represses ghrelin cell specification during pancreas development [3]. Pax4 represses the α-cell transcription factor Arx, which in turn represses Pax4 expression [4]. During pancreas development Nkx6-1 acts as a context dependent transcriptional activator or repressor, activating its own transcription while more broadly repressing gene expression [5,6]. Nkx6-2 is a repressor of Dbx1 in neuronal development [7]. In each of these cases, both in the pancreas and in neuronal development, transcription factor mediated repression of target genes requires the recruitment of cofactors. For many of these transcription factors homologs of Groucho, called the Groucho/Transducin-like enhancer of split (Gro/TLE) family, fulfill this role [7,8].

In Drosophila *groucho *(Gro) acts as a master repressor [9] and regulates transcriptional repression through interactions with Hairy, Hairy related, and Runt family proteins via a WRPW/Y motif, as well as with Dorsal, Engrailed, and Tcf family proteins via an unrelated eh1 (FxIxxIL) motif [10]. Evidence suggests that Gro recruits histone deacetylases (HDACs) that modify the local chromosomal state, silencing gene transcription. There are at least six mouse homologs of Gro (Tle1-6), which all have direct human orthologs. Tle1, 2, 3, 4, and 6 are full-length GRO subfamily members containing a highly conserved WD-repeat domain at their carboxy termini and a glutamine rich (Q) domain at their amino terminal end. Linking these domains is a weakly conserved central region. Tle5 (AES), that lacks much of the central region and the WD-repeat domain, acts as a dominant negative repressor of GRO subfamily members [9-12].

It has been reported that *Tle1 *and *Tle3 *have non-overlapping expression patterns in the brain and spinal cord, while *Tle1*, *2*, and *4 *were shown to be differentially expressed in *in vitro *models of neural and chondrocytic determination [13,14]. Later studies showed that *Tle1*-*4 *have unique but overlapping expression patterns, with the overlaps occurring in putative precursor populations [15]. These results imply that each member of the Gro/TLE family has unique functions in specific cell types, despite the fact they may have similar promiscuous binding affinities for numerous WRPW/Y and eh1 motif containing proteins, and possibly redundant functions in progenitor cell types.

Numerous studies have begun to dissect the roles of the Gro/TLE family members in various developmental systems. To date, Gro/TLE mediated repression has been implicated in pituitary and kidney organogenesis, hematopoiesis, and development of both bone and the eye [16-22]. These studies have revealed a number of pathways and transcriptional repressors utilizing Gro/TLE proteins. For example, all Tcf HMG box transcription factors interact with Tle1, 2, 3, and 4 and repress transactivation in a β-catenin -Tcf reporter gene assay, suggesting a role for the Gro/TLE proteins in Wnt signaling [23]. In addition, Foxa2 (HNF3β) has been shown to interact with Tle1 in Hela and HepG2 cells [24]. The majority of work, however, has focused on understanding the function of the mammalian Gro/TLE proteins in neuronal development. Notch signaling acts to restrict the neural potential of progenitors by antagonizing expression of pro-neural genes. Both Tle1 and Tle2 interact directly with Hes1 and are vital to it's repressive abilities [25-27]. Tle1 also interacts with BF-1 (Foxg1), a regulator of neuronal differentiation [28], mediating the interaction between BF-1 and Hes1 [28]. Several homeodomain transcription factors (i.e. Nkx6-1, Nkx6-2, and Nkx2-2) important in pancreas development are also essential to patterning neuronal specification in the ventral neural tube and interact with Gro/TLE proteins [8,29,30]. A cross-repressive model whereby these transcription factors repress genes involved in the specification of opposing cell types has been proposed [7]. The Gro/TLE family members are a vital component of this model and ectopic expression of Tle5, a dominant negative inhibitor of Gro/TLE mediated repression, perturbs neural tube patterning by extending the expression domains of both *Nkx2-2 *and *Nkx6-1 *[8].

In this work we describe the expression of Gro/TLE family members utilizing nine SAGE libraries created from the developing and adult pancreas. Additionally we identify, and assess the expression of, 34 different transcription factors that contain a Gro/TLE interaction motif in these libraries, and further demonstrate that at least 13 of these are expressed in the endocrine lineage. We then show the dynamic expression of *Tle1 *through *6 *during pancreas development by qRT-PCR. The cell-type specificity of Tle1, 2, 3, and 4 expression was assessed using IHC to co-localize each with Pdx1 at E12.5, Ngn3 at E14.5, Pdx1, Nkx2-2, Insulin, Glucagon, Pancreatic polypeptide and Somatostatin at E18.5, and Insulin and Glucagon in the adult. We then focus on Tle2, as it was expressed widely in endocrine pancreas development, and show that it can interact with Nkx2-2, Nkx6-1, Hes1 and Arx which are all essential in proper endocrine pancreas development. Finally, we demonstrate that Tle2 can modulate the repressive abilities of Arx and that this modulation is dependent upon Arx's Gro/TLE interaction domain.

## Results

### Identification of Gro/TLE proteins and putative Gro/TLE interacting proteins in the developing pancreas

We first assessed the expression of the Gro/TLE family members in nine Serial Analysis of Gene Expression (SAGE) libraries generated from the developing and adult pancreas [31,32]. This data revealed the differential expression of *Tle1 *through *Tle6*, with high levels of both *Tle5 *and *Tle6*, at various stages of pancreas development (Table 1). No tags unambiguously map to Tle4 so it could not be assessed. Since the Gro/TLE proteins interact with transcription factors containing an eh1 or WRPW/Y domain we assessed the expression of the 34 factors present in our pancreas SAGE libraries. Included in this group are several factors essential for proper endocrine cell specification and maturation such as *Hes1, Foxa2*, *Nkx2-2*, and *Pou3f4 *(*Brn4*) (Table 1). *Arx *and *Nkx6-1*, which are essential to proper α- and β-cell development respectively, and contain an eh1 domain, could not be detected as *Arx *does not produce an unambiguous tag while *Nkx6-1 *does not contain a NlaIII site and thus does not produce a SAGE tag.

**Table 1:** Counts of unambiguously identified Gro/TLE family members and eh1 or WRPW domain containing transcription factors in nine pancreas SAGE libraries

Tag	Position^Φ^	Accession	Symbol	Pdx1 GFP+ E10.5 306588	Pdx1 GFP+ E11.5 317716	Ngn3 GFP- E12.5 308745	Ngn3 GFP+ E12.5 320473	Ngn3 GFP+ E13.5 320473	Ngn3 GFP+ E14.5 301222	Whole E14.5 98189	Whole E18.5 81130	Islets Adult 99968	S^□^
Gro/TLE family members													

TGAACAAGCCTGACAAG	2	NM_011599	*Tle1*										
ATGCAGAGCGCCACAGA	16	NM_011599	*Tle1*										
AGAGCTGCGTGCTGTCA	2	NM_019725	*Tle2*	0	0	1	0	5	2	0	0	0	0.5
GAGAGCAGCAATGTGGA	1	NM_009389	*Tle3*	0	0	0	0	0	0	0	0	1	0.0
-	-	NM_010347	*Tle4*	-	-	-	-	-	-	-	-	-	-
TACTATGAGATGTCCTA	1	NM_010347	*Aes*	0	0	3	5	11	7	0	0	0	0.2
GGGTGTGCAGATCCCGA	1	NM_053254	*Tle6*	1	3	2	1	20	9	0	2	0	4.4

Transcription factors containing an eh1 or WRPW Domain													

-	-	NM_007492*	*Arx*	-	-	-	-	-	-	-	-	-	0.04
GGAGTTTTCAAGCACTG	1	NM_010132	*Emx2*	2	1	0	0	0	0	0	0	0	0.09
TTAACGACAAAAAAAAA	1	NM_008259	*Foxa1*	0	3	0	0	0	0	0	0	0	11.46
GTGAAATCCAGGTCTCG	1	NM_010446	*Foxa2*	0	4	0	12	12	8	4	4	8	4.08
TAGTTTTAACAGAAAAC	2	NM_010446	*Foxa2*	0	1	0	0	3	2	0	0	0	3.85
CTGCTATGCACCAAGAT	1	NM_008260	*Foxa3*	0	0	1	1	3	1	0	0	0	0.03
GACTTGTTTTTAAATGT	4	NM_008592	*Foxc1*	1	0	0	0	0	0	0	0	0	0.28
GCTTTGTACAGTAGATG	1	NM_013519	*Foxc2*	3	0	0	0	0	0	0	4	0	0.09
GCCACGCACCAGCCCCT	1	NM_010425	*Foxd3*	0	0	0	0	0	0	0	0	1	0.13
CACTGCATTTGTATATA	1	NM_008235	*Hes1*	0	1	2	0	1	0	0	2	0	13.61
CCGCTGCGTGAGGGTAG	1	NM_019479	*Hes6*	32	3	5	52	13	20	1	0	1	0.04
TTGGCTTCTCTTCCAGT	1	NM_010420	*Hesx1*	0	0	0	0	0	0	0	0	0	0.32
TATATAGCATTACTTCT	2	NM_008245	*Hhex*	0	1	1	1	4	1	0	1	0	4.68
CTAAATTATTATTTAGC	1	NM_019944	*Hlxb9*	0	0	0	0	0	1	0	0	2	4.03
AGCATTCAGATTGCTGA	2	NM_019944	*Hlxb9*	0	0	0	0	2	0	0	0	0	0.47
CTGGTGCTTCACCAAGG	1	NM_010835	*Msx1*	10	1	15	0	0	0	0	0	0	68.93
TACACGTTCTGACAACT	1	NM_010919	*Nkx2-2*	1	1	0	6	14	9	0	1	2	6.98
GAACTCAGCGGAGAAAG	1	NM_008699	*Nkx2-3*	1	0	13	1	0	0	6	0	0	0.05
CTGGCCGCCTTCAAGCC	1	NM_008700	*Nkx2-5*	0	0	0	0	0	0	0	2	0	0.09
CGGGGAGGCTGTCCCCA	3	NM_008700	*Nkx2-5*	0	0	1	0	0	0	0	0	0	-
-	-	NM_144955*	*Nkx6-1*	-	-	-	-	-	-	-	-	-	1.95
CCCCGACGCCAGCCGCC	4	NM_183248	*Nkx6-2*	2	7	0	1	2	0	0	0	0	0.13
GCTGCCTTTGAATAAAG	1	NM_008780	*Pax1*	3	0	0	0	0	0	0	0	0	1.92
TTCTTAATGAGGTCAGC	3	NM_008781	*Pax3*	1	0	0	0	0	0	0	0	0	0.02
CCTAAAGATTCCGCCGC	1	NM_008900	*Pou3f3*	3	0	0	0	0	0	0	0	0	54.12
TGCAAGCTGAAACCGCT	1	NM_008901	*Pou3f4*	1	1	0	38	3	7	0	0	0	0.05
CAGGAGAAACCCTGAGA	1	NM_009323	*Tbx15*	0	0	0	0	0	1	0	0	0	0.37
GCAGCAGCGGCCGCCGC	1	NM_009324	*Tbx2*	0	0	5	0	0	0	0	0	0	0.77
AAACAGGTGTTAAAAAC	1	NM_020496	*Tbx20*	0	0	0	0	0	0	0	1	0	5.94
ACTGTCTCCTCGGGCCT	4	NM_009327	*Tcf1*	0	1	0	1	4	1	0	1	0	0.68
TTAAGACACATCTCTGG	1	NM_009330	*Tcf2*	0	1	0	0	3	1	0	0	0	0.54
CCCACCAGGCCCTCCCC	2	NM_009332	*Tcf3*	9	2	3	1	1	0	0	0	0	0.63
ACCAGGCCTGGAGTTCT	1	NM_009332	*Tcf3*	7	1	1	1	2	0	0	0	0	0.24
GGACAGATGTGAAAAGG	5	NM_013685	*Tcf4*	6	1	5	1	3	0	3	1	1	0.05
GTCAGATTTTTTTTGGA	1	NM_013685	*Tcf4*	1	1	1	2	3	0	1	1	1	35.34
GGACAGAGAGACCCAAG	1	NM_021901	*Tlx1*	0	0	1	0	0	0	0	0	0	0.04

^Φ ^Tag position is calculated by assigning the 3' most CATG in the transcript as the position 1 tag and the next 3' most CATG the position 2 tag etc.

*Tags counts are shown as tags per hundred thousand. This indicates the total number of times a given SAGE tag appears in the library per hundred thousand tags and is used to normalize for libraries of varying size

^□ ^S is the specificity of the tag. Specificity is calculated as described in the materials and methods.

*No unambiguous tags exist for Nkx6-1 or Arx although their expression within the pancreas is well established.

**Figure 1:**
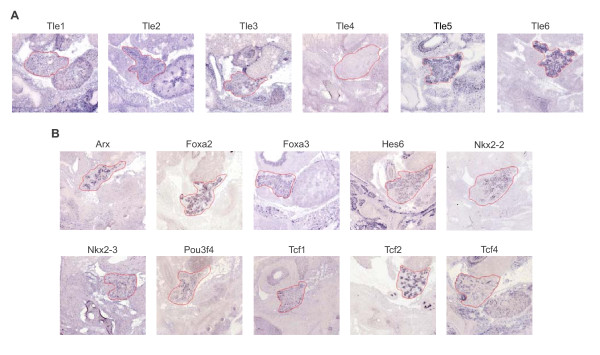
**Expression of Gro/TLE family members and transcription factors with a Gro/TLE interaction domain in the developing pancreas**. Images of *in situ *hybridization staining patterns for **(A) **Gro/TLE family members and **(B) **transcription factors with a Gro/TLE interaction domain on E14.5 whole embryo sagittal sections were obtained from the *GenePaint *website and magnified to show the pancreas (outlined in red).

We then used the *GenePaint * database to assess the staining patterns of these genes in the E14.5 pancreas. *Tle 2 *and *3 *both showed modest staining throughout the epithelium and mesenchyme, while *Tle1 *staining was found predominantly in the mesenchyme (Figure 1A and Table 2). Expression of *Tle4 *could not be detected at this time point in the pancreas. *Tle5 *and *6 *on the other hand showed strong staining throughout the pancreas epithelium. Informative stain was obtained for 22 of the 34 transcription factors containing an eh1 or WRPW/Y domain detected in our SAGE libraries and 11 of these showed stain in the pancreas (Figure 1B and Table 2). The remaining 11 factors for which informative staining was produced but no stain was seen in the pancreas typically showed counts only in the Pdx1 GFP+ libraries or the E12.5 Ngn3 GFP-, including *Foxa1*, *Foxc1*, *Pax1*, *Pax3*, and *Tbx2*, or in libraries representing later developmental stages, including *Foxd3*, *Nkx2-5*, *Tbx20*, and *Tlx1*. Data from the literature also provided evidence of the expression of an additional four of these factors, including *Hes1 *[33], *HlxB9 *[34], *Nkx6-1 *[35], and *Nkx6-2 *[36], which are not in the *GenePaint *database. Thus, in addition to our SAGE data, there is independent evidence demonstrating the expression of thirteen different eh1 or WRPW/Y transcription factors (*Arx*, *Foxa2*, *Foxa3*, *Hes1*, *Hes6*, *Hhex*, *HlxB9*, *Nkx2-2*, *Nkx6-1*, *Nkx6-2*, *Pou3f4*, *Tcf1*, and *Tcf2*), many of which have well established roles in endocrine pancreas development.

**Table 2:** Classification of Gro/TLE family members and eh1 or WRPW domain containing transcription factors into the five expression domains in the developing pancreas using the GenePaint database

Symbol	GenePaint Id#^o^	Trunk	Tip	Epithelium	Vasculature	Mesenchyme	Not detected^#^
Gro/TLE family members							

*Tle1*	MH424					M^	
*Tle2*	MH2206			M		M	
*Tle3*	MH392			M		M	
*Tle4*	EB1917						Y
*Tle5*	N/A						
*Tle6*	MH1997			S			

Transcription factors containing an eh1 or WRPW Domain							

*Arx*	MH740	S					
*Emx2*	N/A						
*Foxa1*	MH864						Y
*Foxa2*	MH515			M			
*Foxa3*	EH531			M			
*Foxc1*	ES2315						Y
*Foxc2*	N/A						
*Foxd3*	MH620						Y
*Hes1*	N/A						
*Hes6*	MH945	M		W			
*Hesx1*	N/A						
*Hhex*	EB22			W			
*Hlxb9*	N/A						
*Msx1*	N/A						
*Nkx2-2*	EN1299	M					
*Nkx2-3*	EB1495					M	
*Nkx2-5*	MH1017						Y
*Nkx6-1*	N/A						
*Nkx6-2*	N/A						
*Pax1*	EN1275						Y
*Pax3*	ES1828						Y
*Pou3f3*	N/A						
*Pou3f4*	MH930	M					
*Tbx15*	EH1284						Y
*Tbx2*	EG1241						Y
*Tbx20*	EG2245						Y
*Tcf1*	EB1413		W	W			
*Tcf2*	MH809			S			
*Tcf3*	MH770						Y
*Tcf4*	MH934					M	
*Tlx1*	EH430						Y

^o ^The accession number of the gene in the GenePaint database , N/A indicates that the gene is not present in the database or that the sections containing informative stain in the pancreas were not present

^ W indicates weak staining, M indicates moderate staining, and S indicates strong staining

^#^Not detected indicates that the probe for the gene produced staining in the embryo, suggesting the probe was functional, but no staining was seen in the pancreas

### qRT-PCR analysis of Gro/TLE genes in pancreas development

**Figure 2:**
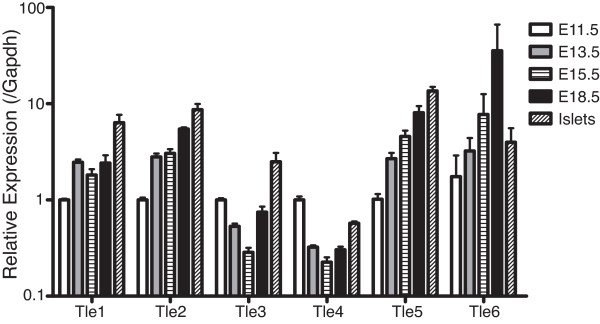
**Relative expression of Gro/TLE family members in the developing pancreas and adult islets**. Expression of *Tle1*, *Tle2*, *Tle3*, *Tle4*, *Tle5 *and *Tle6 *was assessed by qRT-PCR in E11.5 (used as reference), E13.5, E15.5, and E18.5 whole pancreases, as well as adult islets. The data shown is an average of the results obtained from pancreases from four separate litters (pancreases from an individual litter were pooled), each with duplicate reactions or from four separate islet extractions. Error bars indicate the standard deviation of the three averaged replicates.

To determine the overall expression profiles of these genes in pancreas development we assessed their expression by qRT-PCR from E11.5 through to adult islets (Figure 2). The expression profiles of *Tle1 *and *Tle2 *were highly similar, and transcripts for both generally became more abundant as development progressed with peak expression in the adult islets. *Tle3 *expression dropped from E11.5 to E15.5 and was increased again at E18.5 and in adult islets. *Tle4 *expression also dropped at E13.5 and stayed low at the remaining time points analyzed, although levels in the adult islets were higher than those found at E13.5 to E18.5. *Tle5 *expression increased in abundance with development and highest levels were found in adult islets. *Tle6 *expression was almost inverted compared with the *Tle3 *and *Tle4 *profiles, with peak expression at E18.5 and relatively low levels in the islet and E11.5 samples.

### Expression of Gro/TLE proteins at the start of the secondary transition

**Figure 3:**
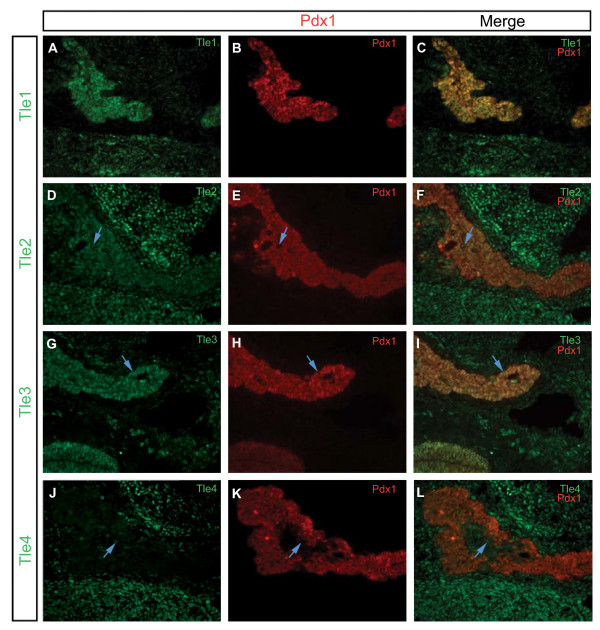
**Expression of Tle1, Tle2, Tle3 and Tle4, as compared to Pdx1 expression, in the E12.5 pancreas**. Transverse sections of E12.5 pancreases were analyzed for the expression of **(A) **Tle1, **(D) **Tle2, **(G) **Tle3 or **(J) **Tle4 (green) in relation to **(B, E, H, K) **Pdx1 expression (red). Merged images are shown in **(C, F, I L)**. Blue arrows indicate cells that are Gro/TLE + but not Pdx1+. Sections were photographed at ×200.

To determine the cell type specificities of Tle1-4 we first analyzed their co-expression with Pdx1, whose expression marks the first appearance of cells capable of giving rise to the endocrine and exocrine pancreas as well as the duodenum [37,38] (Figure 3 and Table 3). It was not possible to obtain Tle5 or Tle6 protein expression data since there are currently no suitable antibodies for these proteins. At this time point (E12.5) Tle1, 2, and 3 were expressed throughout the pancreatic epithelium and all Pdx1 positive cells were also positive for these three Gro/TLE proteins. A few Tle2 and Tle3 positive cells in the pancreatic epithelium were Pdx1 negative. These cells likely correspond to cells that have undergone early differentiation into glucagon positive cells. Tle4 was absent from the pancreatic epithelium and did not co-localize with Pdx1. All four of these Gro/TLE proteins were expressed in the mesenchyme surrounding the developing pancreas, with Tle1 and Tle3 expressed in a few such cells, while Tle2 and Tle4 were widely expressed.

**Table 3:** Summary of co-localization results of Tle1, 2, 3, and 4 with Pdx1 on E12.5 embryonic pancreas tissue

	Pdx1+	Pdx1-	Mesenchyme
Tle1+	Y^o^	N^o^	Y
Tle2+	Y	Y	Y
Tle3+	Y	Y	Y
Tle4+	N	N	Y
Tle1-	N	Y	Y
Tle2-	N	N	Y
Tle3-	N	N	Y
Tle4-	Y	N	Y

^o^Y implies the indicated cell type was found, N indicates the cell type was not found

### Expression of Gro/TLE proteins at the end of the secondary transition

**Figure 4:**
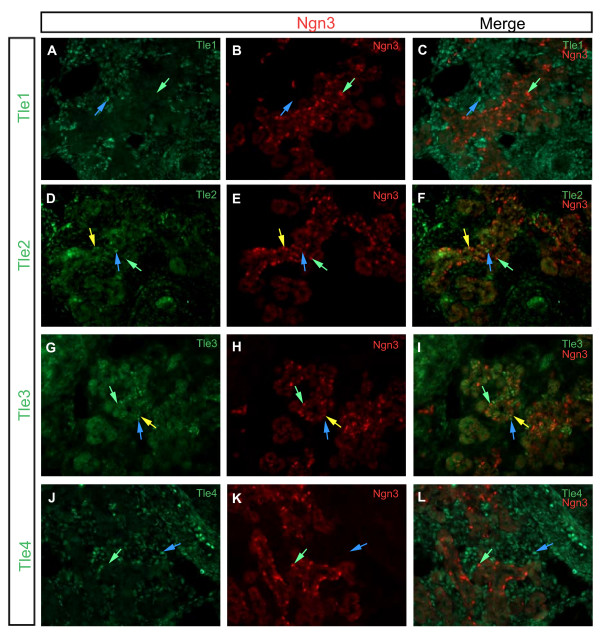
**Expression of Tle1, Tle2, Tle3 and Tle4, as compared to Ngn3 expression, in the E14.5 pancreas**. Transverse sections of E14.5 pancreases were analyzed for the expression of **(A) **Tle1, **(D) **Tle2, **(G) **Tle3 or **(J) **Tle4 (green) in relation to **(B, E, H, K) **Ngn3 expression (red). Merged images are shown in **(C, F, I L). **Blue arrows indicate cells that are Gro/TLE + but not Ngn3+. Yellow arrows indicate cells where the Gro/TLE proteins and Ngn3 co-localize. Green arrows indicate cells expressing Ngn3 only. Sections were photographed at ×200.

**Figure 5:**
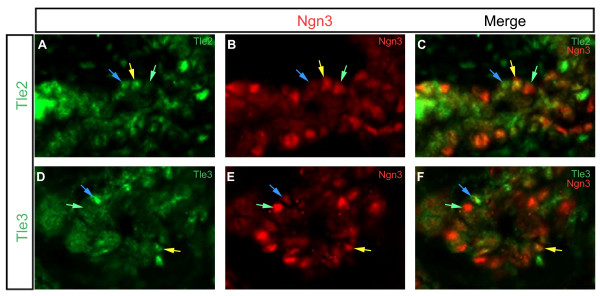
**Close-up of the expression of Tle2 and Tle3, as compared to Ngn3 expression, in the E14.5 pancreas**. Transverse sections of E14.5 pancreases were analyzed for the expression of **(A) **Tle2 or **(D) **Tle3 (green) in relation to **(B, E) **Ngn3 expression (red). Merged images are shown in **(C, F)**. Blue arrows indicate cells that are Gro/TLE + but not Ngn3+. Yellow arrows indicate cells where the Gro/TLE proteins and Ngn3 co-localize. Green arrows indicate cells expressing Ngn3 only. Sections are shown at ×400.

During the secondary transition endocrine progenitors lose expression of Notch signaling components, such as Hes1, and begin to express the bHLH factor Ngn3. In neuronal development Tle1 expression is likewise lost during neural specification of progenitors. To see if a similar process occurs in the pancreas we co-localized Tle1-4 with Ngn3 in the E14.5 developing pancreas. Unlike at E12.5 where Tle1-3 were expressed throughout the pancreatic epithelium these factors clearly showed more restricted expression at E14.5 (Figure 4 and Table 4). At this time point Tle1 was absent from the pancreatic epithelium. Tle2 and Tle3 on the other hand both showed scattered expression through the epithelium. Also, both Tle2 and Tle3 co-localized with Ngn3 in a subset of Ngn3 positive cells. Interestingly, cells that were Ngn3 and Tle2 or Tle3 double positive, cells that were Ngn3 positive and Tle2 and Tle3 negative, and cells that were Ngn3 negative and Tle2 and Tle3 positive, were found (Figure 5). Tle4 was again absent from epithelial cells. Tle3 was only found in a restricted set of mesenchymal cells as observed at E12.5, while Tle1, 2, and 4 were widely expressed in the mesenchyme.

**Table 4:** Summary of co-localization results of Tle1, 2, 3, and 4 with Ngn3 on E14.5 embryonic pancreas tissue

	Ngn3+	Ngn3-	Mesenchyme
Tle1+	N^o^	N	Y^o^
Tle2+	Y	Y	Y
Tle3+	Y	Y	Y
Tle4+	N	N	Y
Tle1-	Y	Y	Y
Tle2-	Y	Y	Y
Tle3-	Y	Y	Y
Tle4-	Y	Y	Y

^o^Y implies the indicated cell type was found, N indicates the cell type was not found

### Expression of Gro/TLE proteins at E18.5, after the secondary transition

Beyond being implicated in controlling progenitor cell maintenance, Gro/TLE proteins also play a role in the maturation of a wide variety of specialized cell types. As previously mentioned many of the transcription factors that play significant roles in proper endocrine cell specification and maturation contain a Gro/TLE interaction domain. To determine which Gro/TLE family members are co-expressed with, and thus might interact with, these factors we co-localized the expression of Tle1 through 4 with Pdx1, Nkx2-2, Insulin, Glucagon, Pancreatic polypeptide, and Somatostatin, which between them mark the four predominant endocrine cell types in the pancreas, at E18.5.

**Figure 6:**
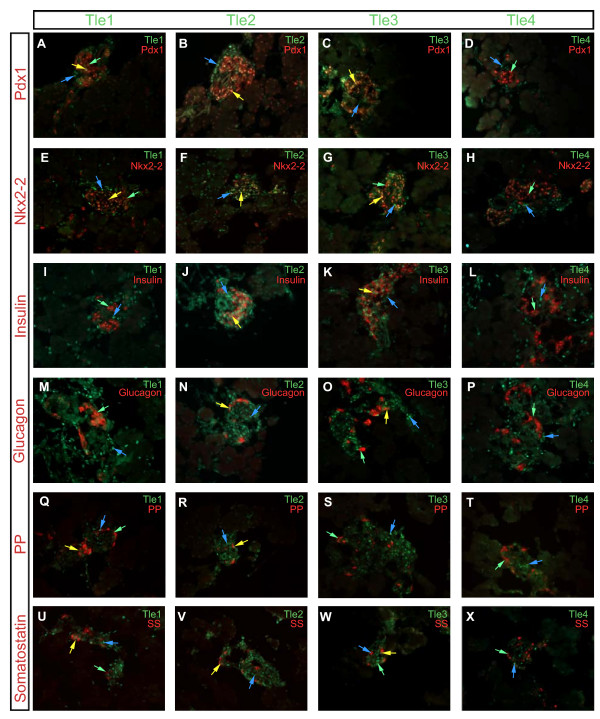
**Expression of Tle1, Tle2, Tle3 and Tle4, in relation to Pdx1, Nkx2-2, Insulin, Glucagon, Pancreatic polypeptide (PP), and Somatostatin expression, in the E18.5 pancreas**. Saggital sections of E18.5 pancreases were analyzed for the expression of **(A, E, I, M, Q, U) **Tle1, **(B, F, J, N, R, V) **Tle2, **(C, G, K, O, S, W) **Tle3 or **(D, H, L, P, T, X) **Tle4 (green) in relation to **(A, B, C, D) **Pdx1, **(E, F, G, H) **Nkx2-2, **(I, J, K, L) **Insulin, **(M, N, O, P) **Glucagon, **(Q, R, S, T) **PP, or **(U, V, W, X) **Somatostatin expression (red). Blue arrows indicate cells that are Gro/TLE + but without co-localization, while yellow arrows indicate cells where the Gro/TLE proteins co-localize with Pdx1, Nkx2-2, Insulin, Glucagon, PP, or Somatostatin as appropriate, and green arrows indicate cells that express Pdx1, Nkx2-2, Insulin, Glucagon, PP, or Somatostatin, as appropriate, in the absence of Gro/TLE protein. Sections were photographed at ×200.

Tle1 was found only in a few endocrine cells and its expression partially over-lapped only with Pdx1, which at this time point is restricted to β and δ cells. Tle1 expression was not seen in either Insulin or Glucagon positive cells. However it was found in cells that stained for the δ cell marker Somatostatin as well as in cells that stained for Pancreatic polypeptide (PP) (Figure 6). Tle2 was abundant in endocrine cells and all Pdx1 and Nkx2-2 positive cells were also Tle2 positive. Tle2 was also found in Insulin, Glucagon, Somatostatin and PP positive cells. Tle3 was nearly as broadly expressed as Tle2 and was found in all Pdx1 positive cells, and most Nkx2-2 positive cells. Tle3 was also found in Insulin, Glucagon, and Somatostatin positive cells but not in PP positive cells. Tle4 was found in rare endocrine cells, none of which expressed Pdx1 or Nkx2-2. Tle4 was also not found in Insulin, Glucagon, PP, or Somatostatin positive cells (Figure 6). A summary of these results can be found in Table 5.

**Table 5:** Summary of co-localization results of Tle1, 2, 3, and 4 with Pdx1, Nkx2.2, Insulin, Glucagon, Pancreatic polypeptide (PP), and Somatostatin (SS) on E18.5 embryonic pancreas tissue

	Pdx1+	Pdx1-	Nkx2-2+	Nkx2-2-	Ins+	Ins-	Glu+	Glu-	PP+	PP-	SS+	SS-
Tle1+	Y^o^	Y	Y	Y	N^o^	Y	N	Y	Y	Y	Y	Y
Tle2+	Y	Y	Y	Y	Y	Y	Y	Y	Y	Y	Y	Y
Tle3+	Y	Y	Y	Y	Y	Y	Y	Y	N	Y	Y	Y
Tle4+	N	Y	N	Y	N	Y	N	Y	N	Y	N	Y
Tle1-	Y	Y	Y	Y	Y	Y	Y	Y	Y	Y	Y	Y
Tle2-	N	Y	N	Y	N	Y	N	Y	N	Y	N	Y
Tle3-	N	Y	Y	Y	N	Y	Y	Y	Y	Y	Y	Y
Tle4-	Y	Y	Y	Y	Y	Y	Y	Y	Y	Y	Y	Y

^o^Y implies the indicated cell type was found, N indicates the cell type was not found

### Expression of Gro/TLE proteins in the adult

**Figure 7:**
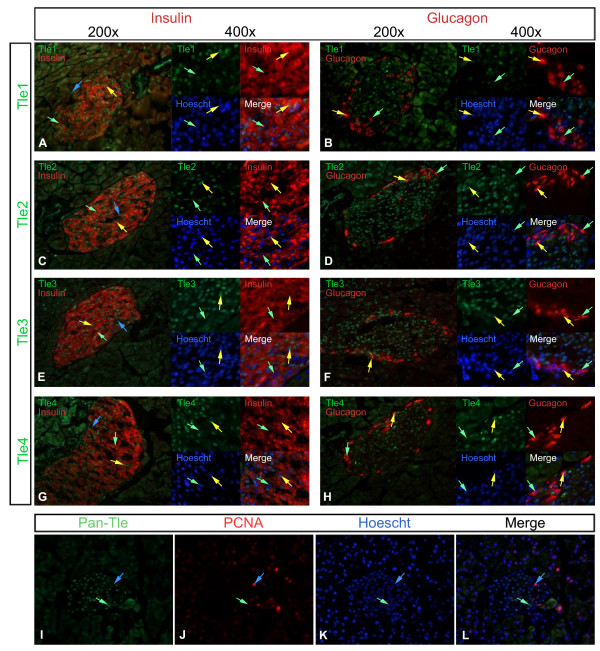
**Expression of Tle1, Tle2, Tle3 and Tle4, in relation to insulin and glucagon expression, in the adult pancreas**. Saggital sections of adult pancreases were analyzed for the expression of **(A, B) **Tle1, **(C, D) **Tle2, **(E, F) **Tle3 or **(G, H) **Tle4 (green) in relation to **(A, C, E, G) **insulin or **(B, D, F, H) **glucagon expression (red). Merged images are shown at 200× and images highlighting regions containing cells that are insulin or glucagon positive but Gro/TLE negative are shown both merged and with the channels separated at 400x. In **(I) **staining with a pan-TLE antibody in relationship to **(J) **PCNA is shown, **(K) **Hoescht staining shows the location of nuclei, and **(L) **shows the merged image. Blue arrows indicate cells that are Gro/TLE + but not insulin+ or glucagon +, while yellow arrows indicate cells where the Gro/TLE proteins and insulin or glucagon co-localize, and green arrows indicate cells that express insulin or glucagon only. Sections were photographed at ×200.

Our qRT-PCR data suggests that the expression of Tle1, 2, and 3 peaks in the adult islets, while expression of Tle4 is almost as high as seen at E11.5. To determine if these genes show cell type specificity of expression within adult islets we next co-localized them with Insulin and Glucagon. Tle1, 2, 3 and 4 expression was quite broad within islets cell types, although Tle4 staining was noticeably weaker. Each of these proteins co-localized with Insulin in the majority of Insulin positive cells. However, in each case a number of cells positive only for Insulin were identified (Figure 7). All of the Glucagon positive cells were also Tle2 and Tle3 positive (Figure 7). The majority of Glucagon positive cells were also Tle1 and/or Tle4 positive. Thus clearly Tle1, 2, 3, and 4 are expressed in both of the major hormone producing cell types in the islet (Table 6). To characterize the Insulin or Glucagon positive Gro/TLE negative cells further we co-localized the Gro/TLE's using a pan-TLE antibody with PCNA, a marker of proliferating cells. All PCNA positive cells showed no or very weak Gro/TLE staining. Several Gro/TLE negative PCNA negative cells were also identified. We also assessed the co-localization of Tle1 through 4 with dolichos biflorus agglutinin (DBA) that marks ductal cells (data not shown). Only Tle2 and Tle3 were found in DBA positive cells. In acinar cells expression of all four of the Tle proteins was found, although Tle4 was present in only a very few cells.

**Table 6:** Summary of co-localization results of Tle1, 2, 3, and 4 with Insulin, Glucagon and DBA on adult pancreas tissue

	Ins+	Ins-	Glu+	Glu-	Ductal (DBA+)	Acinar
Tle1+	Y^o^	Y	Y	Y	N^o^	Y
Tle2+	Y	Y	Y	Y	Y	Y
Tle3+	Y	Y	Y	Y	Y	Y
Tle4+	Y	Y	Y	Y	N	Y
Tle1-	Y	Y	Y	Y	Y	Y
Tle2-	Y	Y	N	Y	N	Y
Tle3-	Y	Y	N	Y	N	Y
Tle4-	Y	Y	Y	Y	Y	Y

^o^Y implies the indicated cell type was found, N indicates the cell type was not found

### Interactions between Tle2 and transcription factors essential to proper pancreas development

**Figure 8:**
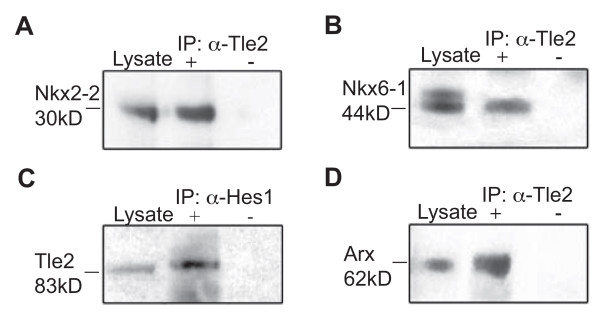
**Interaction of Tle2 with selected factors**. Immunoprecipitations were performed by incubating 200 ug of Min6 cellular lysate with antibodies against Tle2 (+) or IgG (-). Lysate lanes containing whole cell extracts were included as a western blot control. Blots were probed with antibodies against endogenously expressed **(A) **Nkx2-2 or **(B) **Nkx6-1. **(C) **Immunoprecipitations using antibodies against Hes1 (+) or IgG (-) with subsequent blotting with antibodies against Tle2. This was done since the Hes1 band was obscured by the IgG heavy chain band in anti-Tle2 immunoprecpitations with subsequent anti-Hes1 blotting. In **(D) **an Arx cDNA containing a C-terminal 6xHis tag was transiently transfected into Min6 cells, due to the lack of endogenous Arx expression in these cells, 48 hrs prior to the IP and the resulting immunopreciptate probed with an anti-6xHis antibody.

Based on our expression profiling results Tle2 and Tle3 are the most likely candidates to be functional co-repressors for critical endocrine cell transcriptional repressors such as Nkx2-2, Nkx6-1, and others. In support of this, Tle3 interacts with Nkx2-2 in the pancreas [3] and knocking in a mutant Nkx2-2 containing the activation domain but not the Gro/TLE interaction domain produces a phenotype similar to the Nkx2-2 knock out [3] demonstrating that this domain is critical to proper Nkx2-2 function. However, the transcription factors that Tle2 interacts with in the developing pancreas are not known. Thus to determine if Tle2 interacts with transcription factors critical to key stages in pancreas development we used co-immunoprecipitation in the Min6 β-cell line. We first attempted to determine if Tle2, like Tle3, can interact with Nkx2-2 that is essential for the proper specification and maturation of α, β, and PP cells. Anti-Nkx2-2 blots of material immunoprecipitated using an anti-Tle2 antibody produced an ~30 kDa band that was not seen in controls (rabbit IgG used in immunoprecipitation) but was found in western blots of whole cell lysate (Figure 8A). Nkx6-1 has, like Nkx2-2, been shown to interact with Tle4 in *in vitro *binding assays [8]. Nkx6-1 is critical for proper β-cell development and is known to act generally as a transcriptional repressor [5]. We therefore wanted to determine if Tle2 could interact with Nkx6-1. Anti-Tle2 blots of material immunoprecipitated using an anti-Tle2 antibody produced an ~44 kDa band that was not seen in controls but was found in western blots of whole cell lysate (Figure 8B). Interestingly, the expected 46 kDa Nkx6-1 band could only be detected in the western blot [39]. Next, we attempted to determine if Tle2 interacts with Hes1, a critical mediator of Notch signaling in pancreas development [40], which controls pancreas progenitor maintenance and specification [41-43]. Anti-Tle2 blots of material immunoprecipitated using an anti-Hes1 antibody produced an ~83 kDa band (Figure 8C). Recently Arx was shown to have the ability to convert both mature and developing β-cells into cells with an β-cell phenotype [44]. Since it interacts with Tle1 [45] we attempted to determine if it also interacts with Tle2. As Arx is not normally expressed in β-cells we co-transfected a 6xHis tagged Arx expression vector with a Flag tagged Tle2 expression vector. Anti-His blots of material immunoprecipitated using an anti-Tle2 antibody produced an ~63 kDa band that was not seen in controls but was found in western blots of whole cell lysate (Figure 8D). In sum these results indicate that Tle2 can interact with Nkx2-2, Nkx6-1, Hes1, and Arx.

**Figure 9:**
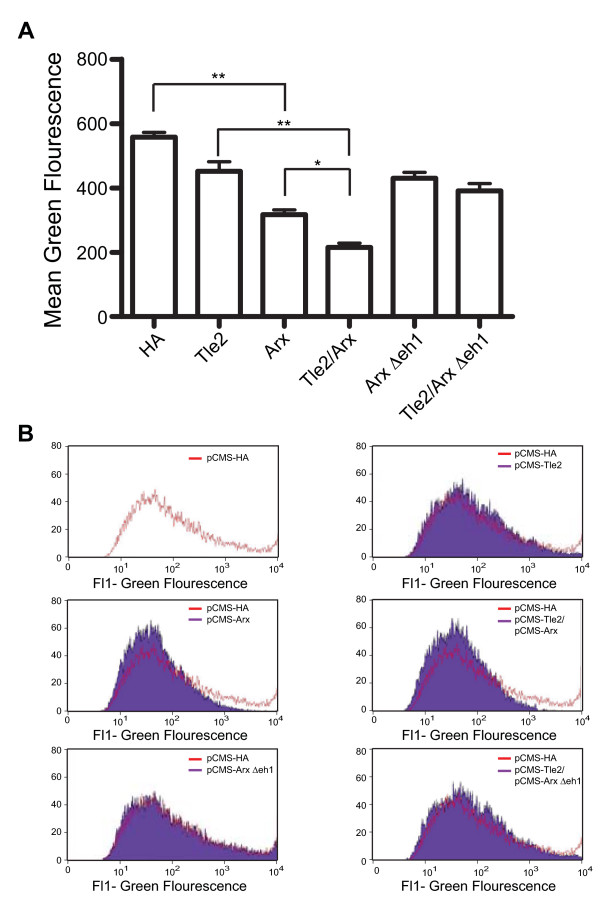
**Tle2 enables Arx mediated insulin reporter suppression**. Min6 cells were transfected with a Rat Insulin Promoter (RIP) EGFP reporter and with either pCMS-HA (HA) vector, pCMS-Flag-Tle2 (Tle2), pCMS-Arx (Arx), pCMS-Flag-Tle2 and pCMS-Arx (Tle2/Arx), pCMS-Arx Δeh1 (Arx Δeh1), or pCMS-Flag-Tle2 and pCMS-Arx Δeh1 (Tle2/Arx Δeh1). The average of four independent experiments is shown in **(A) **and representative histograms from one experiment in **(B)**. Statistical analysis was carried out using a one way ANOVA with a Bonferroni Multiple Comparison post test to compare means. * p < 0.01 and ** p < 0.001 (n = 4 experiments with triplicate samples in each experiment).

Since Arx is able to convert mature β-cells into cells with an α-cell phenotype, we hypothesized that Gro/TLE-Arx interactions might be important to this ability. To assess this possibility we used a rat insulin promoter (RIP) reporter driving the expression of Enhanced Green Fluorescent Protein (EGFP). Transfection of the RIP-EGFP reporter with a Tle2 expression vector modestly reduced the green fluorescence intensity (Figure 9). However, transfection of the RIP-EGFP reporter with an Arx expression vector resulted in a 43% (p < 0.001) reduction in the mean green fluorescence intensity produced by the reporter as compared to transfection with the empty vector. Co-transfection of Arx with Tle2 resulted in a 61% (p < 0.001) reduction in the mean green fluorescent intensity produced by the reporter as compared to transfection with empty vector, a significant reduction from transfection with Arx alone (p < 0.01). Transfection with a Δeh1 Arx construct, which lacks the Gro/TLE interaction domain, resulted in a modest repression of the mean green fluorescence and co-transfection of this vector with the Tle2 vector did not alter the level of repression seen.

## Discussion

Our qRT-PCR and IHC data indicate that the Gro/TLE proteins are expressed throughout pancreas development and suggest that they may act at multiple stages of pancreas development. Interestingly, we see the expression of all four Gro/TLE factors in the pancreatic mesenchyme. In our *GenePaint *analysis we identified two transcription factors with a Gro/TLE interaction domain expressed in the mesenchyme, *Nkx2-3 *and *Tcf4*. Tcf4 can interact with Gro/TLE proteins [23] although the significance of these, and any interactions between Nkx2-3 and Gro/TLE proteins, in the pancreatic mesenchyme remains to be elucidated. Tle1, 2, and 3 were expressed throughout the epithelium in the pancreas prior to the secondary transition, while at E18.5 only Tle2 and Tle3 were broadly expressed in the various developing endocrine cell types. We identified 13 different transcription factors also expressed in the endocrine lineage that contain Gro/TLE interaction domains. Many of these factors have known roles in pancreas development including *Hes1 *that is essential for proper endocrine specification, as well as *Foxa2*, *Nkx2-2*, *Nkx6-1*, and *Pou3f4 *(*Arx*) that are essential for proper endocrine cell maturation. The presence of these putative interacting transcription factors that are essential to various steps of pancreas development in our SAGE libraries further supports our hypothesis that the Gro/TLE proteins play a role during multiple phases of pancreas development.

At the initiation of pancreas development (~E8.5) pancreatic epithelial precursor cells express *Pdx1*, although scattered Pdx1 negative Glucagon positive cells can be found. The co-expression of Tle1, 2 and 3 at E12.5 agrees with previous studies indicating that Gro/TLE expression overlaps in progenitor cell populations in other tissues. During neuronal development it has been suggested the primary role of the Gro/TLE factors is to maintain cells in the progenitor state through interactions with Hes1 [46], and they likely play this same role in the pancreas at this time.

The differentiation of pancreatic progenitors into the various endocrine precursor cell types is under the control of Notch signaling, via lateral inhibition [47]. During this phase of pancreas development (E12.5–E14.5), called the secondary transition, committed endocrine progenitor cells are defined by their expression of *Neurogenin 3 *(*Ngn3*) [38] whose expression is otherwise repressed by Hes1 [47]. At this time point we identified Ngn3 negative Tle2/3 positive cells that likely represent duct cell precursors generated from pancreatic progenitors in which active Notch signaling has repressed *Ngn3 *activation. In addition, we find Ngn3 Tle2/3 double positive cells that showed generally dim *Ngn3 *staining, suggestive of precursors in which an endocrine fate has yet to be fully established. Last we find Ngn3 bright cells that are Tle2/3 negative and likely represent committed endocrine precursors. As Tle1 and Tle4 are not expressed in the epithelium at this time, these cells represent cells in which none of the traditional Gro/TLE co-repressor factors are present, indicating that the belief that these factors are ubiquitous throughout pancreas development is incorrect. Regardless, the wide expression of Tle1, 2, and 3 prior to the secondary transition, and the obvious disappearance of Tle2 and Tle3 with concomitant appearance of Ngn3, suggests that these factors play a role in Notch mediated lateral inhibition. Our demonstration that Tle2 can interact with Hes1 in pancreas cells, as it does in neural development [27], provides additional support for this hypothesis.

By E18.5 Tle2 and Tle3 expression are clearly reactivated and both were expressed broadly in endocrine cell types, in agreement with our qRT-PCR data. Our E18.5 co-localization data showed that Tle1 is expressed in Pdx1 (that marks β and δ cells), Nkx2-2 (that marks α, β, and PP cells), PP, and Somatostatin positive cells, but not in Insulin or Glucagon positive cells suggesting that it is expressed in δ and PP cell types, but not in α or β cells. Tle2 co-localized with all the markers tested, indicating that is expressed in α, β, δ and PP cell types. Tle3 co-localized with all of the markers tested with the exception of PP, and in agreement with this, Nkx2-2 positive Tle3 negative cells were found, indicating that Tle3 is expressed in α, β, and δ cell types but not in PP cells. Tle4 was the least abundant of the four Gro/TLE family members assessed and co-localized with none of the markers tested suggesting Tle4 may be expressed specifically in ghrelin producing ε cells, although this remains to be confirmed.

The obvious co-expression of the Gro/TLE proteins in islet cells, as was found in pancreatic progenitors at E12.5, is intriguing. Also of interest is the presence of rare Gro/TLE negative, Insulin positive cells. Gro/TLE proteins have been implicated in controlling the cell cycle [48] and we show that some of these Gro/TLE negative cells represent replicating β-cells. The expression and roles of these factors in ductal and acinar cells is also of interest. It is clear that more work needs to be done to further assess the roles of these factors in these cell types.

In general our IHC data agree with the relative expression levels identified by qRT-PCR. In the qRT-PCR data, expression of *Tle1 *and *2 *increased with development. At first glance this may seem in disagreement with the IHC data that indicates these factors decrease in expression in the epithelium. However, it is worth noting that they are still widely expressed in the surrounding mesenchyme. *Tle3*, on the other hand, was not expressed widely in the mesenchyme and its expression clearly does drop at E13.5 to E15.5 and is then reactivated at E18.5, as was found in the IHC data. Expression of all four of the Gro/TLE family members analyzed here was relatively high in the adult islets, again in agreement with the IHC data. Interestingly, the qRT-PCR expression profiles of *Tle1*-*Tle4 *are quite different from that of *Tle5 *and *6*. Additionally from our *GenePaint *analysis Tle5 and Tle6 are both abundantly expressed throughout the pancreatic epithelium at E14.5. It is striking to note that these data suggest that *Tle1*, *2*, and *3 *are being shut off in Ngn3 positive endocrine precursors at roughly this time point. Tle5 is a dominant negative repressor of Tle1-Tle4 [9-12]. Likewise in neuronal development Tle6 (Grg6) antagonizes Tle1 interactions with BF-1 inhibiting BF-1 target gene repression and inducing neural progenitors to differentiate [49]. It is therefore likely these factors are acting to limit or prevent Gro/TLE mediated target repression during pancreas development and our expression data suggests this may play an important role during the secondary transition.

Arx can repress *Pax4 *[4,50] and likely other β-cell specification genes and convert β-cells and β-cell progenitors into cells with an α-cell phenotype [44]. As this effect includes the loss of insulin expression we used a rat promoter insulin reporter construct to determine if Arx mediated repression of β-cell transcripts is Gro/TLE dependent. We found that co-expression of Arx and Tle2 enhanced repression of the insulin reporter as compared to Arx alone. However, this repression was not seen using an Arx construct lacking the eh1 domain. These data suggest that the repression of β-cell transcripts by Arx, which in turn leads to the repression of insulin and other genes characteristic of β-cells, is at least in part Gro/TLE dependent.

**Figure 10:**
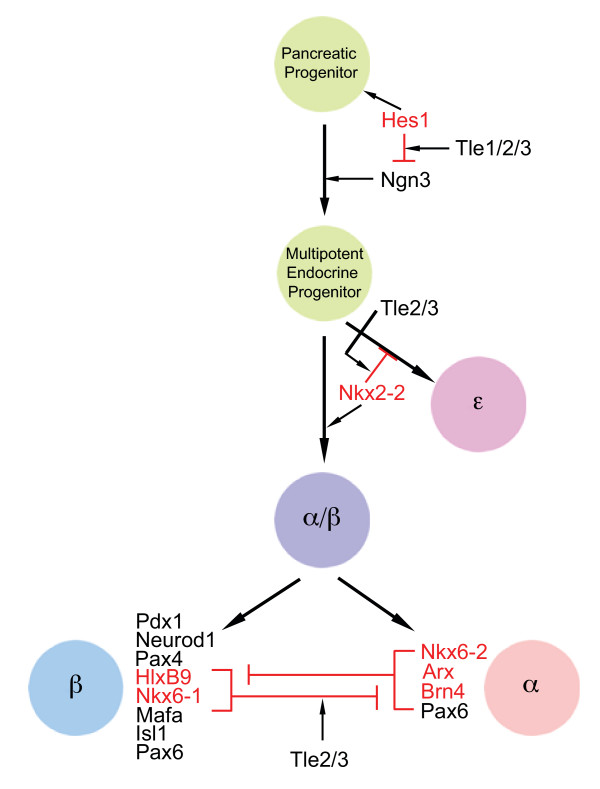
**A model of the proposed role of the Gro/TLE factors in cell fate choices during endocrine pancreas development**. A schematic depicting a cross-repressive model of the genetic network regulating α-, β-, and ε-cell fate is shown based on our experimental data, as well as evidence from the literature on pancreas development [3,47] and on models of neuronal cell specification.

Our results agree with studies on neuronal development that have defined a dual role for Gro/TLE factors in first controlling the differentiation of progenitor cell types and then later in the specification and maturation of specific cell types [46]. Our co-immunoprecipitation data demonstrates that Tle2 is able to interact with Nkx2-2, Nkx6-1, Hes1 and Arx, which are key controllers of endocrine cell specification and maturation. Further our IHC data indicates that Tle2 and Tle3, in particular, co-localize with these factors in pancreas development suggesting these interactions occur *in vivo*. Based on our results we propose a model for Gro/TLE action in the developing pancreas (Figure 10). In this model Tle1, 2, and 3 initially play a redundant role in enacting the repression of Hes1 target genes, such as *Ngn3*, thereby maintaining the cells in a progenitor state. In cells that begin to express higher *Ngn3 *levels, *Hes1 *expression is lost and cells differentiate into multipotent endocrine progenitors. Subsequently tripotent α/β/ε precursors develop into an ε (ghrelin producing) precursor or an α/β bipotent precursor based on repression of the ε cell fate by Nkx2-2 [3]. Experimental evidence has demonstrated that Gro/TLE interactions are critical to this process and both Tle2 and 3 are co-expressed with Nkx2-2 and can interact with it [3]. Subsequently, α/β-cell precursors undergo α or β cell specification wherein we hypothesize that pro-β-cell eh1 motif containing factors repress α-cell activating factors while pro-α-cell eh1 motif containing factors repress α-cell activating factors, several of which do so in a putatively Gro/TLE dependent fashion. The specific targets of these genes, in general, are not known.

This model is, in part, based on the action of many of these same pancreas-essential transcription factors in neuronal development in which the interactions of cross repressive transcription factors and Gro/TLE proteins are critical for the proper inhibition of opposing factors [7,8]. Evidence suggesting that this model applies to pancreas development is beginning to emerge. For example, Nkx2-2 knock-out mice develop normal numbers of endocrine precursors which differentiate almost exclusively into ghrelin cells. Knock-in of a mutant Nkx2-2 composed of a DNA binding domain and a Gro/TLE interaction domain, but lacking an activation domain results in the development of primarily α-cells [3]. In contrast knocking in a mutant Nkx2-2 containing the activation domain but not the Gro/TLE interaction domain produces a phenotype similar to the Nkx2-2 knock out. These data suggest that repression of pro-ghrelin cell factors via Gro/TLE proteins is essential for the appropriate differentiation of a hypothetical tripotent (α, β, and ghrelin cell) precursor into a bipotent α/β cell precursor; whereas activation of target genes by Nkx2-2 is required for the proper differentiation of α/β progenitors into β-cells. In addition, Nkx6-1 and Nkx6-2 act in a cross repressive fashion during motor neuron development [7,30,51] and at least in the case of Nkx6-1 this repression is Gro/TLE dependent [8]. The expression of Nkx6-2 in the pancreas can be seen from E8.5 to E10.5 whereas Nkx6-1 becomes predominantly expressed starting at E11.5 [36] and Nkx6-1 knock-out mice have elevated levels of Nkx6-2 [52]. These data suggest that Nkx6-1 represses Nkx6-2 in a Gro/TLE dependent manner as occurs in motor neuron development [30]. Recent evidence indicates Nkx6-1 does not act to repress alternative cell fates directly [36] and instead acts to "prime" progenitor cells to be able to become β-cells. How the repressive versus activational activities of Nkx6-1 factor into this remains to be elucidated, although it seems likely that Nkx6-1 inhibits the expression of factors that repress pro-β cell factors such as Myt1, whose expression is reduced in Nkx6-1-/- mice [52]. On the other hand, Arx misexpression throughout the developing pancreas induces a clear loss of β and δ cells [44]. In fact, Arx is directly cross-repressive with Pax4 during pancreas development [4], further indicating that this factor represses genes essential to alternative cell fates and here we show that Arx interacts with Tle2 and that Tle2 modulates it's repressive effects.

## Conclusion

At least 13 Gro/TLE interaction domain containing transcription factors are expressed in the endocrine pancreas lineage. These factors are known to control multiple critical steps in pancreatic development including progenitor cell specification as well as endocrine cell type specification and maturation. Tle1, 2, 3, and 4 are expressed dynamically during pancreas development. Tle1, 2, and 3 overlap in expression in the pancreatic epithelium at E12.5 suggesting they have redundant roles at this time. During the secondary transition their expression alters dramatically with Tle1 being lost from the epithelium and Tle2 and Tle3 expressed in only a subset of Ngn3 positive cells. This indicates that as Ngn3 expressing cells mature they lose Tle2 and Tle3 expression. However, by E18.5 these factors are re-activated and co-expressed in α, β, and δ cells, although only Tle2 was found in PP cells. Tle1 expressing cells were less abundant and we show Tle1 is expressed in δ and PP cells but not in α or β cells. Tle4 was only found in a rare subset of endocrine cells that did not express any of the markers tested. We also show that Tle2 can interact with factors involved in progenitor cell maintenance (Hes1), and endocrine cell maturation and specification (Nkx2-2, Nkx6-1, and Arx). Moreover, we provide evidence that Tle2 modulates the ability of Arx to repress the β-cell phenotype using a rat insulin reporter in a Gro/TLE interaction domain dependent fashion. In sum these data suggest that Tle1 – 4 are involved in a wide range of processes and are recruited by numerous transcription factors essential to proper pancreas development and function.

## Methods

### Mouse maintenance and islet isolation

All mice were bred and maintained at the British Columbia Cancer Research Centre animal facility according to the guidelines of the Canadian Council on Animal Care. All protocols were approved by the University of British Columbia Animal Care Committee. Mice were housed in microisolator units, provided with Purina mouse food and autoclaved water *ad libidum*, and were maintained at 20°C ± 2°C under a light/dark cycle (light: 05:00–19:00 and dark: 19:00–05:00). Males were mated overnight with up to three females and females were checked for plugs before 9:30 the following morning. Plugged mice were considered to be 0.5 days post coitum (dpc). Islets were purified from 8–10 week old ICR males by collagenase digestion and gradient centrifugation as previously described [53].

### SAGE data analysis

SAGE data was analyzed using DiscoverySpace4 [54]. All SAGE libraries were generated and sequenced as part of the Mouse Atlas of Gene Expression [31] and Mammalian Organogenesis (MORGEN) projects. The data was filtered for sequence quality so that each tag had a 95% or greater probability of being correct, using the PHRED score quality assessment software [55]. Tag to gene mapping was performed using the mouse Refseq, MGC, and Ensembl databases using the DiscoverySpace program. Tags were considered sense position matches if they mapped in the sense orientation to the gene and antisense matches if they mapped in the opposite orientation. A tag was considered unambiguous if it matched a single sense position gene in all of the databases, and ambiguous if it mapped to multiple genes in a sense position regardless of the mapping position. The specificity of tags was determined by first obtaining the counts for the tags in 205 different Mouse Atlas Libraries. From this the mean of the tag counts in all the libraries (M_a_) was determined and compared to tag count in the library of interest (C_i_) to obtain the mean ratio (M_r_). The total number of libraries the tag was found in, or library count, was next determined (L_a_) as was the total counts of the tag in the library under analysis (C_i_). The specificity (S) was then calculated as: S = M_r _log_1.3_(C_i_)/L_a_. Thus tags with a high mean ratio that appear in relatively few libraries and are expressed more abundantly will have the highest specificities.

### Quantitative real-time PCR

Probes for *Tle1*, *2*, *3*, and *4 *as well as *GAPDH *were purchased from Applied Biosystems, Foster City, CA. An ABI 7500 real-time PCR system (Applied Biosystems) and Universal PCR Master Mix (Applied Biosystems) was used for all reactions. Six replicate cDNAs were obtained by reverse transcription (RT) of 1 μg of total RNA from newly isolated embryonic pancreas tissue or from Min6 cells for each RT. 10 ng of generated cDNA was used in each reaction with all reactions done in duplicate. Samples were normalized to *Gapdh*, and the fold increase compared to E11.5 pancreas or untreated Min6 cells (as appropriate) was calculated using 2^-ΔΔCt ^[56].

### GenePaint Analyses

Images of *in situ *hybridization staining patterns for whole embryo sagittal sections were obtained from the *GenePaint *website (Visel et al., ). Higher magnification images of the area of the embryo containing the pancreas were obtained and the pancreas outlined. The brightness and contrast of some of the images was altered using Photoshop to better assess the staining pattern. Genes were then classified as showing trunk, tip, epithelial, mesenchymal or vasculature staining [57].

### Immunohistochemistry

Immunohistochemistry was performed on E12.5, E14.5, E18.5 embryonic, or adult, pancreas cryo-sections sections using the following antibodies: 1/100 dilution of guinea pig anti-Insulin (Stem Cell Technologies Inc.); 1/500 dilution of guinea pig anti-Glucagon (Linco); 1/100 dilution of guinea pig anti-PP (Millipore); 1/1000 dilution of mouse anti-Somatostatin (Santa Cruz); 1/100 dilution of mouse anti-Nkx2-2 (Developmental Studies Hybridoma Bank); 1/100 dilution of mouse anti-Ngn3 (Developmental Studies Hybridoma Bank); 1/10,000 dilution of guinea pig anti-Pdx1 (kindly provided by Christopher Wright); 1/1000 dilution of rabbit anti-Tle1 (kindly provided by Stefano Stifani); 1/1000 dilution of rabbit anti-Tle2 (Santa Cruz); 1/1000 dilution of rabbit anti-Tle3 (Santa Cruz); 1/1000 dilution of rabbit anti-Tle4 (kindly provided by Stefano Stifani); 1/100 dilution of rabbit anti-pan TLE (Santa Cruz); 1/100 dilution of mouse anti-PCNA. Primary antibodies were detected using a 1/2000 dilution of Alexa 488 or Alexa 546 conjugated anti-rabbit, anti-mouse, anti-goat or anti-guinea pig (Invitrogen) antibodies as appropriate.

### Cell Culture and Co-Immunoprecipitation

Min6 cells were maintained in high glucose (4,500 mg/l) Dulbecco's Modified Eagle's Medium (DMEM; StemCell Technologies Inc.) supplemented with 10% fetal calf serum and 5 mM L-glutamine. Immunoprecipitations were performed on 3 × 10^6 ^Min6 cells or Min6 cells transfected with pCMS-6xHis-Arx and pCMS-Flag-Tle2, as appropriate. Cells were lysed and pre-cleared with 20 uL ProteinA-Sepharose (Rockland) to reduce background. Supernatants were incubated with 10 ug Rabbit Anti-Tle2 at 4° for 2 hours, and then with 20 uL of a 50% ProteinA-Sepharose slurry for 1 hour. Beads were washed three times in lysis buffer, resuspended in SDS sample buffer and electrophoresed on a 10% poly-acrylamide gel. Proteins pulled down using an anti-Tle2 (Santa Cruz) or anti-Hes1 (Santa Cruz) antibody were immunoblotted onto Immobilon-P membranes (Millipore) and detected with mouse anti-Nkx2-2 (Developmental Studies Hybridoma Bank), mouse anti-Nkx6-1 (Developmental Studies Hybridoma Bank), rabbit anti-Tle2 (Santa Cruz), or rabbit anti-His (Abcam). Negative controls to assess the specifity of the pulldowns included: rabbit IgG pulldowns and lysate only controls.

### Rat Insulin Reporter Assays

Assays were performed by transfecting 2 × 10^5 ^Min6 cells with 600 ng of the appropriate vectors using Lipofectamine (Invitrogen) (pCMV-HA was used to adjust total DNA amounts) using the following amounts as appropriate: 300 ng of rat insulin promoter EGFP reporter plasmid, 50 ng of pCMS-Arx, 50 ng of pCMS-Arx Δeh1, 200 ng of pCMS-Flag-Tle2. 48 hrs after transfection the cells were harvested and single cell-suspensions prepared. Cells were then stained with 7-AAD (5 μg/ml) and analyzed by flow cytometry using a FACSCalibur flow cytometer and CELLQuest software (BD Pharmigen). Cells were gated based on the forward- versus side-scatter profile and on the 7-AAD versus forward scatter profiles to gate for viable cells, and a pCMS-HA only transfected sample was used to determine appropriate gating. The mean green fluorescence intensity was then determined for the samples using the CELLQuest software (BD Pharmingen).

## Authors' contributions

BH performed all IHC, SAGE data analysis, and GenePaint analysis; assisted with the qRT-PCR studies and RIP assays, and wrote the manuscript. BZ performed co-immunoprecipitation experiments and assisted with the qRT-PCR studies. MB performed the Rat insulin promoter assays. CH is the senior author. All authors have read and approved the manuscript.
